# Supplementary material for: Classification of tauopathies from human brain homogenates through salt‐modulated tau amplification
Source: Alzheimers Dement. 2026 Feb 13;22(2):e71112. doi: 10.1002/alz.71112 (PMC12902886; doi:10.1002/alz.71112)
Supplement: Supplementary file 1 — Supporting Information [file ALZ-22-e71112-s002.docx]

**SUPPLEMENTARY INFORMATION**

**Classification of Tauopathies from Human Brain Homogenates through Salt-Modulated Tau Amplification**

Alessia Santambrogio^1*^, Michael A. Metrick II^1,2,3^*, Peifeng Xu^1^, Nicholas C. T. Gallagher^1^, Shunsuke Koga^4,5^, Bernardino Ghetti^6^, Dennis W. Dickson^4^, Byron Caughey^2^ and Michele Vendruscolo^1,+^

*^1^Centre for Misfolding Diseases, Yusuf Hamied Department of Chemistry,*

*University of Cambridge, Cambridge, UK*

*^2^Laboratory of Neurological Infections and Immunity, Rocky Mountain Laboratories, Division of Intramural Research, National Institute for Allergy and Infectious Diseases, Hamilton, Montana, USA*

*^3^Departments of Pathology and Laboratory Medicine,*

*University of California at San Francisco, California, USA*

*^4^ Department of Neuroscience, Mayo Clinic, Jacksonville, Florida, USA*

*^5^Department of Pathology and Laboratory Medicine,*

*The University of Pennsylvania, Pennsylvania, USA*

*^6^Department of Pathology, Indiana University School of Medicine,*

*Indianapolis, Indiana, USA*

*Authors contributed equally to this manuscript

+Correspondence: [mv245@cam.ac.uk](mailto:mv245@cam.ac.uk)

**Supplementary Table** **1**. Clinical, neuropathologic, and RT-QuIC diagnostic data of samples used in this study.

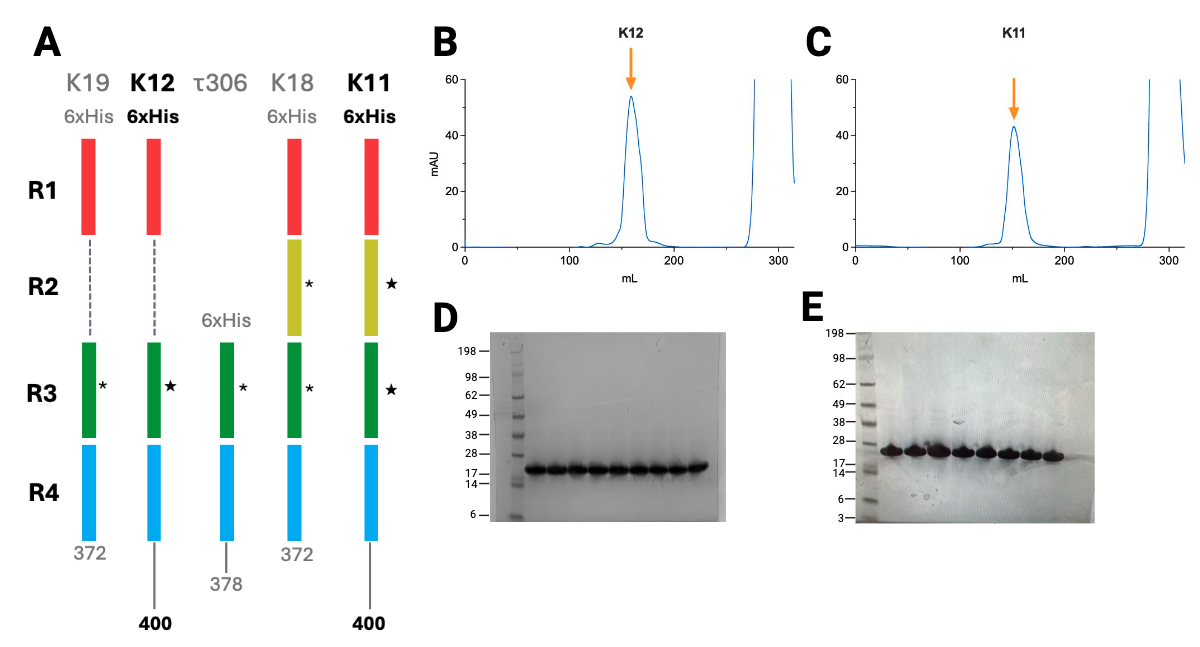


K12

K11

**Figure S1. Sequences and purification of K11 and K12 tau.** (**A**) Schematics of truncated and mutated K11 and K12 RT-QuIC substrates relative to the previously used RT-QuIC substrates. Cysteines in the canonical tau sequences were mutated to serine (*) or alanine (★) for recombinant tau fragments. (**B**)  Size exclusion chromatography (SEC) of K12. (**C**)  SEC of K11. (**D**) Coomassie-stained SDS-PAGE analysis of K12 tau after SEC. (**E**) SDS-PAGE of K11 tau after SEC.

**Figure S2. Optimisation of K11 tau RT-QuIC: selection of sodium salts. (A)** ThT fluorescence traces from 192 replicate spontaneous K11 substrate reactions performed at an ionic strength of 1.5 M in various sodium salts. **(B)** Violin plots of ThT fluorescence maxima illustrating conformational clustering across different salts. Conformer diversity was assessed visually by counting distinct lobes in the distributions; Na_3_citrate n = 4, Na_2_SO_4_ n = 10, NaPi n = 5, NaCl n = 4. **(C)** Box-and-whisker plots showing the distribution of aggregation half-times (t_1/2_) corresponding to the reactions in (**A**). boxes represent inter-quartile range, whiskers represent outer quartiles.

**Figure S3. Optimisation of K11 tau RT-QuIC: Ionic strength titration with sodium sulfate. (A)** Aggregation half-time (t_1/2_) analysis of brain homogenate-seeded K11 reactions across a range of sodium sulfate concentrations, reported as ionic strength (M); boxes represent inter-quartile range, whiskers represent outer-quartiles. **(B)** Fold separation analysis, calculated as the ratio of the fastest t_1/2_ control value divided by tauopathy-seeded t_1/2_ values from (**A**); error bars represent standard deviation; the dashed line indicates the minimum fold separation (1) required to distinguish seeded from spontaneous aggregation. **(C)** Example fold separation calculation for a CBD-seeded reaction, illustrating the division of the fastest control t_1/2_ (40 h) by a representative CBD t_1/2_ at 1.5 M ionic strength. Asterisks in (**C**) mark the corresponding data points shown in (**A**) and (**B**). Brain samples used in this figure include 203, 209, MC24, AD2, and CVD1.

**Figure S4. Optimisation of K11 tau RT-QuIC: substrate concentration and strain discrimination**. (**A, B**) t_1/2_ analysis of brain homogenate-seeded reactions compared to control CVD-seeded reactions in 2 µM K11 (A) and 4 µM K11 (B). Shaded area in **A and B** shows reaction times at which a second replicate of the reactions seeded by CVD (control) exceeded the threshold. (**C,D**) Relative ThT maxima from the reactions in (A) and (B). Asterisks represent statistical significance of one-way ANOVA with multiple comparisons; **** p < 0.0001, *** p < 0.001, ** p < 0.01, * p < 0.1, ns non-significant. Brain samples used in this figure include 203, 209, MC21, MC24, AD2, and CVD1.

**Figure S5. Primary ThT fluorescence data of K12 RT-QuIC endpoint dilution analyses.** Panels show traces from 7 replicate reactions at the designated dilutions of cerebrovascular disease (CVD1), Alzheimer disease (AD6), and Pick disease (PiD6) brain homogenates in 250 mM Na_3_citrate. The fractions in the upper left corner of each panel indicate the ThT-positive/total replicate reactions. (**B**) Violin plots of ThT maxima from reactions in (A) focusing on dilutions between 1x10^-4^ and 1x10^-6^. Statistical significance was assessed by one-way ANOVA; **** p < 0.0001; *** p < 0.001; ns, not significant. (**C**) box-and-whisker plots of t_1/2_ values from reactions in (**A**). Horizontally aligned overlapping points at 80 h represent reactions in which ThT fluorescence remained at baseline levels, indicating no fibrils formed; boxes represent inter-quartile range, whiskers represent outer quartiles.

**Figure S6. Primary ThT fluorescence data of K11 RT-QuIC endpoint dilution analyses.** Panels show traces from 14 replicate reactions at the designated dilutions of cerebrovascular disease (CVD1) and 30 repllicates for corticobasal degeneration (CBD, 203), and globular glial tauopathy type II (GGT type II, MC24) brain homogenates in 250 mM Na_3_citrate. The fractions in the upper left corner of each panel indicate the ThT-positive/total replicate reactions. (**B**) Violin plots of ThT maxima from reactions in (A)**.** Statistical significance was assessed by one-way ANOVA; **** p < 0.0001; *** p < 0.001; ns, not significant. (**C**) box-and-whisker plots of t_1/2_ values from reactions in (**A**). Horizontally aligned overlapping points at 66 h represent reactions in which ThT fluorescence remained at baseline levels, indicating no fibrils formed; boxes represent inter-quartile range, whiskers represent outer quartiles.

**Figure S7. K12 RT-QuIC raw fluorescence traces in 250 mM sodium citrate**. Individual panels represent a singular patient brain assayed at 1x10^-5^ dilution, with 30 replicates per panel.

**Figure S8.** **K11 RT-QuIC raw fluorescence traces**. Traces are shown in 500 mM sodium sulfate (top) and 250 mM sodium citrate (bottom). Individual panels represent a singular patient brain assayed at 1x10^-4^ dilution, with sixteen replicates per panel.

**Figure S9. Serial dilution kinetics of CBD and AGD-seeded K11 tau RT-QuIC reactions**. CBD (203,204) and AGD (MC28, M28) brain homogenates were assayed at 10-fold dilutions between 1x10^-4^ and 1x10^-6^ where each box represents a singular reaction condition, with sixteen replicate reactions per condition. The leftmost twelve reactions were performed in 500 mM sodium sulfate, rightmost twelve reactions in 250 mM sodium citrate; boxes represent inter-quartile range, whiskers represent outer quartiles.
